# Comparison of native myocardial T1 and T2 mapping at 1.5T and 3T in healthy volunteers: Reference values and clinical implications

**DOI:** 10.1007/s00508-018-1411-3

**Published:** 2018-12-05

**Authors:** Marcel Granitz, Lukas J. Motloch, Christina Granitz, Matthias Meissnitzer, Wolfgang Hitzl, Klaus Hergan, Alexander Schlattau

**Affiliations:** 1 Department of Radiology, Paracelsus Medical University, Müllner Hauptstraße 48, 5020 Salzburg, Austria; 2 Department of Internal Medicine II, Paracelsus Medical University, Salzburg, Austria; 3 Research Office (biostatistics), Paracelsus Medical University, Salzburg, Austria

**Keywords:** Cardiac, MRI, Normal, Field strength

## Abstract

**Background:**

Myocardial native T1 and T2 mapping are promising techniques for quantitative assessment of diffuse myocardial pathologies; however, due to conflicting data regarding normal values, routine clinical implementation of this method is still challenging.

**Methods:**

To evaluate this situation during daily clinical practice the characteristics of normal values obtained in 60 healthy volunteers who underwent magnetic resonance imaging (MRI) scans on 1.5T and 3T scanners were studied. The T1 modified look-locker inversion recovery (MOLLI; 5(3)3; modified for higher heart rates) and T2 navigator gated black-blood prepared gradient-spin-echo (GraSE) sequences were used.

**Results:**

While age and body mass index did not affect relaxation times, a gender and heart rate dependency was found showing higher T1 and T2 values in females, whereas at higher heart rates a prolongation of T1 and a shortening of T2 relaxation times was found. Particularly prone to artifacts were T2 measurements at 3T and the inferolateral wall. In the individual setting mean relaxation times for T1 were 995.8 ± 30.9 ms at 1.5T and 1183.8 ± 37.5 ms at 3T and 55.8 ± 2.8 ms at 1.5T and 51.6 ± 3 ms at 3T for T2 indicating a high dependency of reference values on MRI protocol when compared to the literature. Furthermore, as presumed mean T1 and T2 values correlated in the same individual.

**Conclusions:**

The T1 and T2 relaxation times depend on physiological factors and especially on MRI protocols. Therefore, reference values should be validated individually in every radiological institution before implementing mapping protocols in daily clinical practice. Correlation of mean T1 and T2 values in the same proband at both field strengths indicates intraindividual reproducibility.

## Introduction

Cardiac magnetic resonance imaging (CMRI) is the gold standard in noninvasive myocardial tissue characterization [1]; however, early, subtle or diffuse myocardial tissue changes may not be evident using late gadolinium enhancement or conventional scanning techniques which depend on relative signal intensity differences between affected and adjacent, unaffected myocardial tissue [2]. Meanwhile, the use of relaxometry methods has gained widespread acceptance [2–4]. Mapping techniques overcome the dependency on visually distinguishing differences in signal intensities. They allow direct measurement of T1 spin-lattice relaxation time or T2 spin-spin relaxation time in milliseconds and quantify the signal of each voxel on a standardized scale. Relaxation times depend on the water content in the tissue and allow further tissue characterization. Relaxation times vary in different tissues but also within the same tissue undergoing pathophysiological changes (e.g. inflammation, ischemia, edema and fibrosis) [2]. In addition, unenhanced T1 measurements enable the detection of various T1-altering substances, such as lipids in Anderson-Fabry disease, proteins in cardiac amyloidosis or iron deposits in hemochromatosis [5–7]. Furthermore, it was shown that native T1-mapping measurements are significantly predictive of all-cause mortality and heart failure events in nonischemic cardiomyopathy [8].

The T2-weighted (T2w) CMRI and T2-weighted short tau inversion recovery (STIR) techniques are well-established sequences for the detection of myocardial edema [9] to differentiate acute from chronic myocardial changes [10, 11]. The quantitative approach of T2-mapping is a robust, accurate and fast alternative to conventional T2w and STIR sequences for the detection of edema in acute inflammatory cardiomyopathies. This method has the potential to even monitor myocardial inflammation in healing myocarditis [12], which has important clinical implications. Furthermore, it plays a role in the diagnosis of myocardial infarction by overcoming some restrictions of conventional T2w imaging [4], such as sensitivity to myocardial motion, surface coil intensity variation, high subendocardial signal from static blood, incomplete blood suppression and inter-reader or intra-reader variability [13].

An important aspect of mapping techniques is that the obtained information has prognostic relevance. The pathology cannot only be detected but also be quantified and the course of disease can be monitored under treatment. Therefore, mapping techniques have the potential to act as biomarkers to facilitate diagnostic decision making (e. g. iron overload, Anderson-Fabry disease) [14].

Despite promising results in the literature, routine implementation of this technique remains challenging. This might be associated with conflicting data regarding normal values. Recent publications suggest that even in sophisticated facilities, a variety of technical, image acquisition and patient-dependent factors influence T1 and T2 relaxation times [15]. To evaluate this issue the implementation of relaxometry methods in a general radiology department performing approximately 500 CMRI annually was investigated. To obtain reliable standard values for both 1.5T and 3T, the T1 and T2 relaxation times were measured in the same cohort using established state of the art mapping sequences.

## Subjects and methods

### Study population

The study protocol was approved by the local ethics committee of the province of Salzburg (Number 415-EP/73/412-2014). The cohort consisted of 60 healthy volunteers (33 women/27 men), stratified into two equally sized age groups <45 and ≥45 years of age (Table 1). Written informed consent was obtained from all subjects in this study.

**Table 1:** Study population characteristics, values are mean ± SD

	Female	Male	p
Total study cohort	33	27	–
Healthy volunteers^a^	32	26	–
Age (years)	40.1 ± 13.7	42.3 ± 12.5	0.632
BMI (kg/m^2^)	24.1 ± 4.3	23.9 ± 3.7	0.846
Heart rate (bpm)	65.4 ± 10.0	62.5 ± 13.0	0.352
LV end-diastolic volume (ml)	123.0 ± 23.2	148.2 ± 30.1	<0.001
LV end-systolic volume (ml)	43.2 ± 13.6	60.5 ± 20.7	<0.001
LV ejection fraction (%)	65.6 ± 5.8	60.0 ± 6.6	0.001
LV mass (g)	104.8 ± 16.0	139.1 ± 26.2	<0.001
LV mass index (g/m^2^)	58.6 ± 5.9	72.5 ± 9.6	<0.001
RV end-diastolic volume (ml)	122.9 ± 26.1	155.2 ± 28.2	<0.001
RV end-systolic volume (ml)	45.4 ± 13.7	68.4 ± 18.9	<0.001
RV ejection fraction (%)	63.5 ± 6.2	57.4 ± 6.1	<0.001
RV mass (g)	52.8 ± 11.0	71.2 ± 15.0	<0.001
RV mass index (g/m^2^)	29.4 ± 4.6	37.1 ± 6.4	<0.001
Interventricular septal thickness (mm)	8.4 ± 1.3	9.8 ± 1.4	<0.001

*BMI* body mass index, *LV* left ventricular, *RV* right ventricular

^a^two volunteers had to be excluded due to newly detected structural heart disease

Inclusion criteria were no medical history concerning cardiac events, no regular medication, absence of symptoms indicative of cardiovascular conditions and sinus rhythm during the MRI examination. Furthermore, normal cardiac chamber dimensions, normal left ventricular (LV) and right ventricular (RV) wall motion, normal LV and RV ejection fraction as well as normal LV and RV mass were confirmed by the evaluation of CMRI-cine sequences.

Exclusion criteria were general contraindications for MRI (pacemakers, cochlear implants, claustrophobia), pregnancy, consumption of alcohol prior to the examination, known systemic disease and hardness of hearing.

### CMRI protocol

The CMRI procedure was performed using both a commercially available 1.5T and 3T scanner (1.5T Ingenia, 3T Achieva, both Philips Healthcare, Best, Netherlands). All volunteers were consecutively examined on two MRI scanners (1.5T and 3T) in a supine position. A combined 16-channel anterior/posterior coil system was used for the 1.5T scanner, a 6-channel cardiac RF coil and MultiTransmit technology was used for the 3T scanner. At 3T, in addition to B0 shimming, after B1 calibration RF shimming was applied for better field uniformity. Electrocardiography (ECG) was used for cardiac gating.

All studies were performed by experienced radiographers who were trained in the study protocol.

#### Cine imaging

Balanced steady-state free-precession (bSSFP) cine images were obtained in the 4‑chamber view and in a stack of short axes (SAX) covering the ventricles. Wall motion was assessed and the LV ejection fraction was quantified. Imaging parameters for 1.5T were: repetition time (TR) = 2.8 ms, echo time (TE) = 1.38 ms, flip angle (FA) = 60°, field of view (FOV) = 350 × 286 mm^2^, matrix = 176 × 133, slice thickness = 8 mm, SENSE factor = 2 and cardiac phases = 30. Imaging parameters for 3T were: TR = 2.8 ms, TE = 1.4 ms, FA = 45°, FOV = 320 × 350 mm^2^, matrix = 176 × 208, slice thickness = 8 mm, SENSE factor = 2 and cardiac phases = 24.

#### T1 mapping

Data for T1 mapping were acquired in three SAX slices (apical, mid-ventricular and basal). The balanced SSFP-based modified look-locker inversion recovery (MOLLI) technique was used [16]. Imaging parameters for 1.5T were: TR = 2.2 ms, TE = 1.02 ms, FA = 35°, FOV = 380 × 256 mm^2^, voxel size 2 × 2 × 10 mm^3^ and SENSE factor = 2. The MOLLI schema used was 5 beats (3s) 3 beats. Imaging parameters for 3T were: TR = 2.6 ms, TE = 1.3 ms, FA = 35°, FOV = 380 × 356 mm^2^, voxel size 2 × 2 × 10 mm^3^ and SENSE factor = 2. The MOLLI schema used was 5 beats (3s) 3 beats.

#### T2 mapping

Data for T2 mapping were acquired in three SAX planes (apical, mid-ventricular and basal). A navigator gated black blood prepared gradient spin-echo sequence (GraSE) was used [17] and 9 images were acquired. The imaging parameters for 1.5T were: TR = 1 heartbeat, 9 echos, TE1 = 12 ms, ∆TE = 6.2 ms, FA = 90°, EPI factor: 3, FOV = 380 × 380 mm^2^, voxel size: 2 × 2 × 10 mm^3^ and SENSE factor = 2. The imaging parameters for 3T were: TR = 1 heartbeat, 9 echos TE1 = 11 ms, ∆TE = 5.7 ms, FA = 90°, EPI factor: 3, voxel size: 2 × 2 × 10 mm^3^ and SENSE factor = 2.

### Image analysis

#### Evaluation of left and right ventricular function and mass

Using Philips software (Extended Workspace, Philips Healthcare) the bSSFP cine images in SAX and 4CH were evaluated for wall motion abnormalities. The LV and RV ejection fraction were determined by manually contouring the endocardial borders in end-diastolic and end-systolic cardiac phase in SAX. The LV and RV mass, as well as the body surface indexed mass were determined by manually contouring of the endocardial and epicardial borders of both ventricles.

#### T1 and T2 mapping—qualitative assessment

**Fig. 1:**
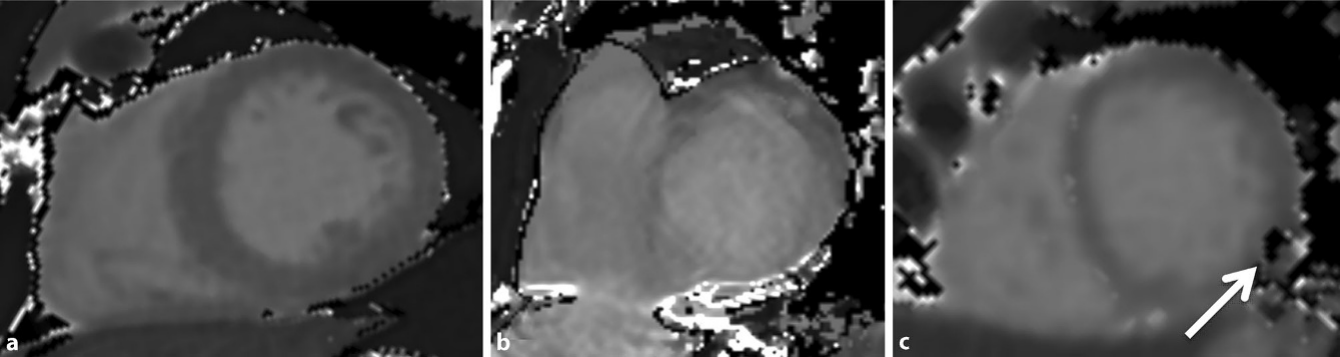
Short axis view of the heart showing T1 mapping artifacts at 3T. Good quality map (**a**), motion artifact obscuring all segments in a slice which is too close to the base (**b**), susceptibility artifact in the inferolateral wall (*white arrow*) (**c**)

All source images were assessed visually regarding artifacts caused by cardiac or respiratory motion or susceptibility. Artifacts were specified as focal, diffuse or band-shaped signal abnormalities of the myocardium (bright/white or dark/black) and led to exclusion of the affected segment. Some myocardial segments had to be excluded due to misplacement of the stack. Examples of artifacts that led to segment exclusions are illustrated in Fig. 1. The quality in consensus was analysed by two board-certified radiologists specializing in cardiac imaging.

#### T1 and T2 mapping—quantitative assessment

**Fig. 2:**
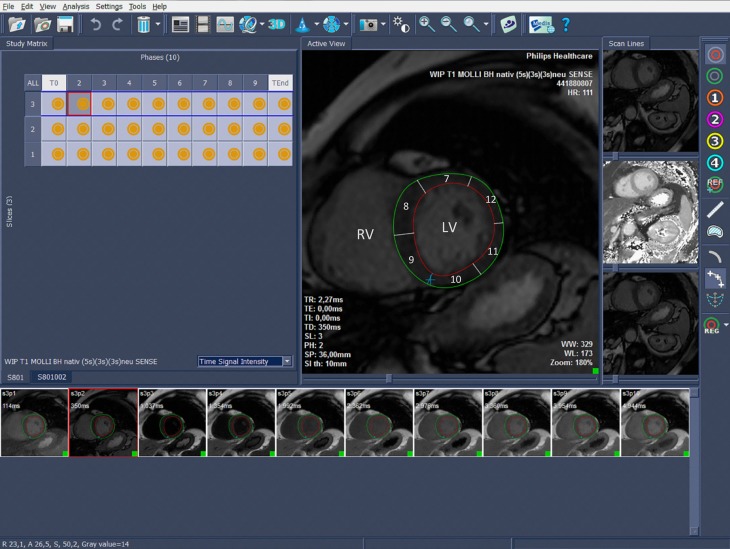
Example of semiautomatic contouring and segmentation of the left ventricular myocardium in a mid-ventricular T1 short axis map. The table on the left shows the active slice (1–3) and number of the inversion time (T0- T9 and TEnd) which is displayed enlarged on the right side. On the right side segmentation according to the AHA 16-segment model is performed. Below, raw images ordered by inversion times are shown. *RV* right ventricle, *LV* left ventricle

The QMASS 7.5-Software (QMASS 7.5 Enterprise solution with T1&T2 mapping add-on, Medis Medical Imaging Systems, Leiden, Netherlands, www.medis.nl) was used for generating the maps, segmentation and segmental quantification. The maps were generated by defining a region of interest (ROI) in the mid-ventricular septum. The endocardial and epicardial contours were manually drawn. To avert disturbances from the blood pool, 10% of the subendocardial and subepicardial aspect of the ROI were excluded automatically (Fig. 2). The myocardium was semiautomatically segmented into a 16 segment bull’s-eye plot according to the American Heart Association (AHA-model, 6 basal segments, 6 mid-ventricular segments and 4 apical segments) [18]. Quantitative assessment was performed by two cardiac-imaging radiologist in consensus.

### Statistical methods

Data were checked for consistency and normality. Means, standard deviations (SD), 95% confidence intervals (CI), Pearson’s correlation coefficient and 1‑factorial ANOVA model together with two-sided, independent t‑tests were used to evaluate data. Normal ranges were estimated by using 2.5% and 97.5% percentiles to end up with 95% CI for the empirical distributions. Regression analysis was used to compare values between 1.5T and 3T. A *p*-value less than 5% indicates a statistical significance. All statistical analyses in this report were performed by use of STATISTICA 13 [19] and were done by one of the authors (WH). Data consistency was checked by analyzing the range of the variables. Data cleansing was done by validating and correcting values against a known list of entities and looking for unexpected or erroneous data entries. Normality was tested by using Kolmogorov-Smirnov tests.

## Results

All 120 CMRI scans were carried out without adverse events. The scan was incomplete in one subject due to technical problems and two subjects had to be excluded due to the detection of a chronic disease (sarcoidosis and an atrial septal defect II, respectively). Therefore, 115 examinations in 58 subjects were available for further analysis. The T1 and T2 maps at 1.5T were available for 57 subjects, T1 and T2 maps at 3T were available for 58 subjects. Due to malpositioning of the basal slice too close to the atrioventricular plane 15 segments (1.6%) had to be eliminated from the T1 measurements at 1.5T, 10 segments (1.1%) from T1 at 3T, 15 segments (1.6%) from T2 at 1.5T and 21 segments (2.3%) from T2 at 3T measurements. Females and males showed no statistically significant differences with respect to age, heart rate and body mass index (Table 1).

### T1 mapping

**Fig. 3:**
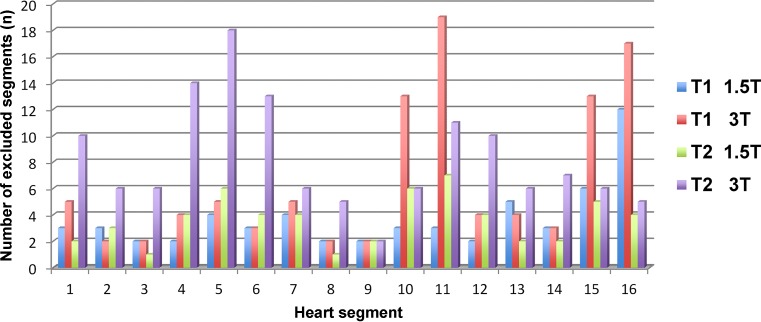
Column graph illustrating the number of excluded segments due to artifacts depending on the individual mapping sequence and the heart segment

At 1.5T a total of 897 segments were available of which 59 segments were excluded due to artifacts (6.6%). Particularly prone to artifacts was segment 16 (apicolateral wall). At 3T a total of 918 segments were available, of which 104 segments were excluded due to artifacts (11.3%). The inferolateral wall in the apical and mid-ventricular slices (segments 10, 11, 15 and 16) was especially prone to artifacts (Fig. 3).

Mean global relaxation times for both field strengths are presented in Table 2. We observed a prolongation of mean global T1 relaxation times at 3T compared to 1.5T by 188 ms (1184 ± 38 ms vs. 996 ± 31 ms).

**Table 2:** Overall cohort, relaxation times in milliseconds for T1 and T2 at 1.5T and 3T

		Segments (*n*)	Mean	Min.	Max.	95% CI	SD
T1	1.5T	838	995.8	916.8	1066.8	987.5–1004.2	30.9
	3T	814	1183.8	1118.9	1287.9	1173.8–1193.9	37.5
T2	1.5T	840	55.8	46.3	62.1	55.0–56.5	2.8
	3T	776	51.6	44.5	61.4	50.8–52.4	3.0

*Min*. minimum, *Max*. maximum, *CI* confidence interval, *SD* standard deviation

Furthermore, the influences of basic volunteer characteristics on relaxation times were examined focusing on gender, age and since controversial data are presented in the literature, also on body mass index (BMI) and heart rate. No statistically significant differences in different age groups or in volunteers with a BMI above 25 kg/m^2^ were found (Table 3).

**Table 3:** Differences in volunteer groups depending on gender, heart rate (HR), BMI (body mass index in kg/m^2^) and age (years). Relaxation times in milliseconds (significant differences, *p* < 0.05, *italics*)

–		**Female**		**Male**		*p*
		*Mean*	*SD*	*Mean*	*SD*	
**T1**	*1.5T*	1004.6	31.5	984.4	26.5	*0.015*
	*3T*	1196.2	38.8	1167.3	28.9	*0.003*
**T2**	*1.5T*	56.7	1.7	54.6	3.5	*0.008*
	*3T*	52.0	3.0	51.0	2.9	0.2
–		**HR** **<** **70**		**HR** **≥** **70**		*p*
		*Mean*	*SD*	*Mean*	*SD*	
**T1**	*1.5T*	989.5	29.4	1011.1	29.7	*0.017*
	*3T*	1176.8	33.3	1203.0	42.7	*0.019*
**T2**	*1.5T*	56.2	2.1	54.8	4.0	0.09
	*3T*	52.1	2.9	50.1	2.9	*0.02*
–		**BMI** **≤** **25**		**BMI** **>** **25**		*p*
		*Mean*	*SD*	*Mean*	*SD*	
**T1**	*1.5T*	998.7	34.1	989.8	22.6	0.321
	*3T*	1187.6	37.5	1175.8	37.3	0.278
**T2**	*1.5T*	55.9	2.8	55.6	3.0	0.731
	*3T*	51.9	3.0	50.9	3.0	0.275
–		**Age** **<** **45**		**Age** **≥** **45**		*p*
		*Mean*	*SD*	*Mean*	*SD*	
**T1**	*1.5T*	991.5	34.6	1000.9	25.5	0.264
	*3T*	1184.1	41.9	1183.5	32.6	0.957
**T2**	*1.5T*	56.5	2.39	55.0	3.15	0.057
	*3T*	51.3	2.31	51.9	3.63	0.442

**Fig. 4:**
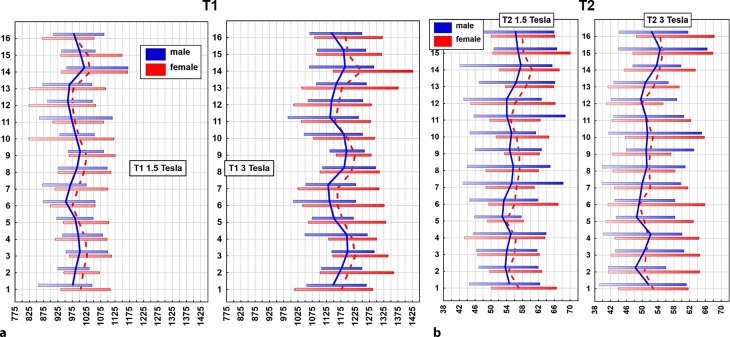
Vertical plots illustrating per segment mean T1 (**a**) and T2 (**b**) relaxation times in milliseconds plus normal ranges (95% CI for distributions): males = *blue line*, females = *red dashed line*, relaxation time in milliseconds on x‑axis, heart segment on y‑axis

**Fig. 5:**
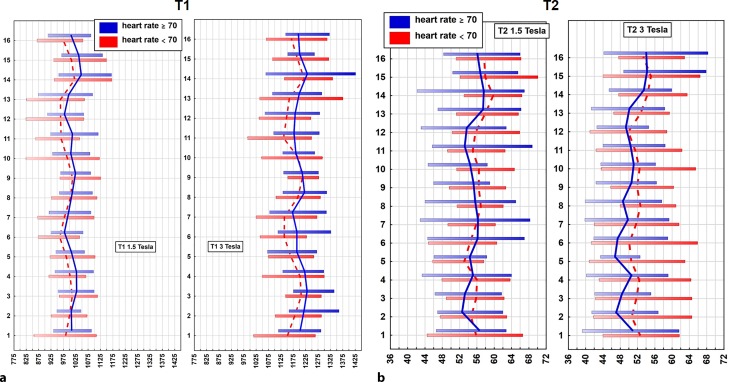
Vertical plots illustrating per segment mean relaxation times in milliseconds plus normal ranges (95% CI for distributions) for T1 (**a**) and T2 (**b**) at both field strengths. Heart rate ≥ 70 bpm = *blue line*, Heart rate <70 bpm = *red dashed line*, relaxation time in milliseconds on x‑axis, heart segment on y‑axis

Gender-specific and heart rate-specific global values are presented in Table 3. As previously discussed (Table 1), there were no significant differences regarding the basic characteristics between females and males; however, global T1 relaxation times were significantly longer in females (a detailed per segment delineation is presented in Fig. 4a). Furthermore, lower heart rates (<70 bpm) were associated with significantly shorter global T1 relaxation times (Fig. 5a).

The next step was focused on slice and segment-specific differences using a 16-segment model of the heart. Table 4 illustrates slice-dependent differences. Generally, T1 relaxation times (independent of field strength) were highest in the apical slice and lowest in the midventricular slice. Whereas T1 values at 1.5T did not show significant slice-dependent differences (*p* = 0.057), at 3T significant differences were detectable between the base and the apical slice (*p* = 0.013) and furthermore between the midventricular and the apical slice (*p* = 0.002).

**Table 4:** Per slice values, all volunteers, relaxation times in milliseconds

	Position	Valid segments (*n*)	Mean	Minimum	Maximum	2.5% percentile	97.5% percentile	SD
T1 1.5T	Base	310	997.0	931.7	1067.3	947.8	1057.5	31.3
	Middle	326	991.1	900.8	1083.2	915.0	1074.3	33.6
	Apex	202	1000.1	877.7	1098.3	919.5	1095.7	40.5
T1 3T	Base	317	1182.3	1106.3	1313.7	1114.7	1273.0	40.9
	Middle	302	1179.6	1100.8	1254.0	1111.0	1251.3	39.9
	Apex	195	1194.4	1099.3	1322.0	1122.8	1299.3	46.1
T2 1.5T	Base	307	54.8	47.3	60.7	47.6	59.9	3.0
	Middle	318	55.5	43.5	62.5	44.9	61.5	3.3
	Apex	215	57.8	47.0	65.7	49.3	64.8	3.6
T2 3T	Base	260	50.4	42.8	63.0	43.1	59.2	4.4
	Middle	308	51.1	42.4	60.0	43.2	59.7	3.3
	Apex	208	53.6	46.9	61.6	47.2	61.0	3.0

**Fig. 6:**
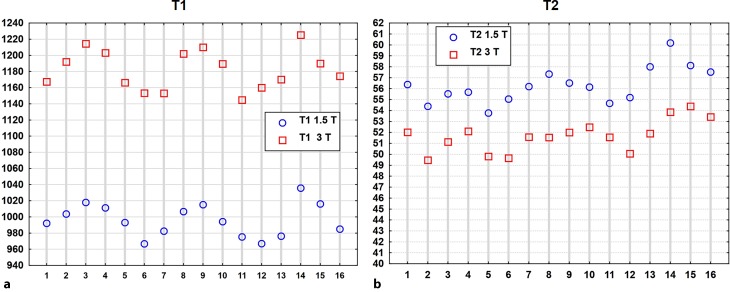
Scatter plots, presenting per segment correlations of all available values for (**a**) T1 (r = 0.92, *p* < 0.001) and for (**b**) T2 relaxation times in milliseconds (r = 0.82, *p* < 0.001) at both field strengths. Heart segment on x‑axis, relaxation time in milliseconds on y‑axis

The T1 values reflect a high per segment correlation at 1.5T and 3T (r = 0.92, *p* < 0.001) showing higher values in the septal and inferior segments and lower values in lateral segments (Fig. 6a).

**Fig. 7:**
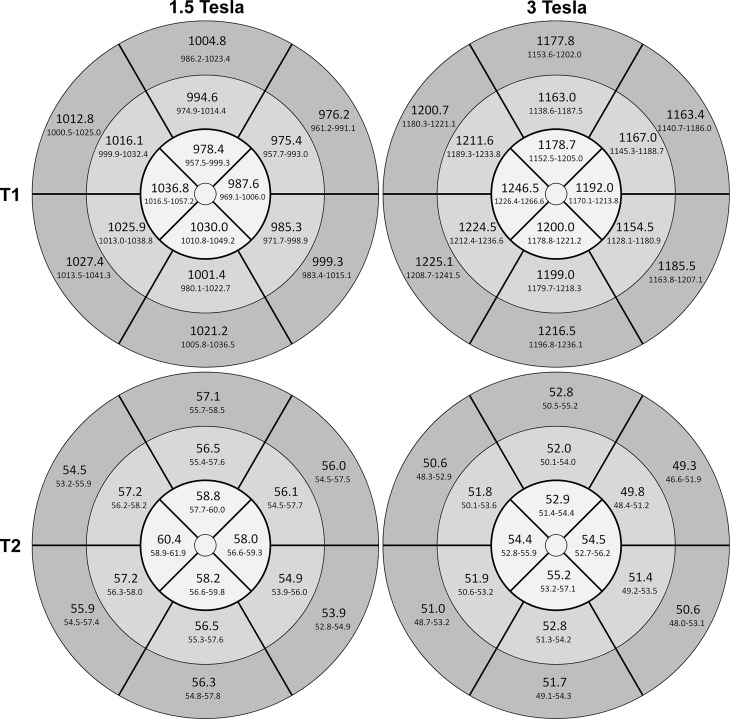
16-segment heart model (AHA), bull’s-eye plot of the left ventricle. Mean T1 and T2 relaxation times in milliseconds per segment plus 95% CI for females at 1.5T (*left*) and at 3T (*right*)

**Fig. 8:**
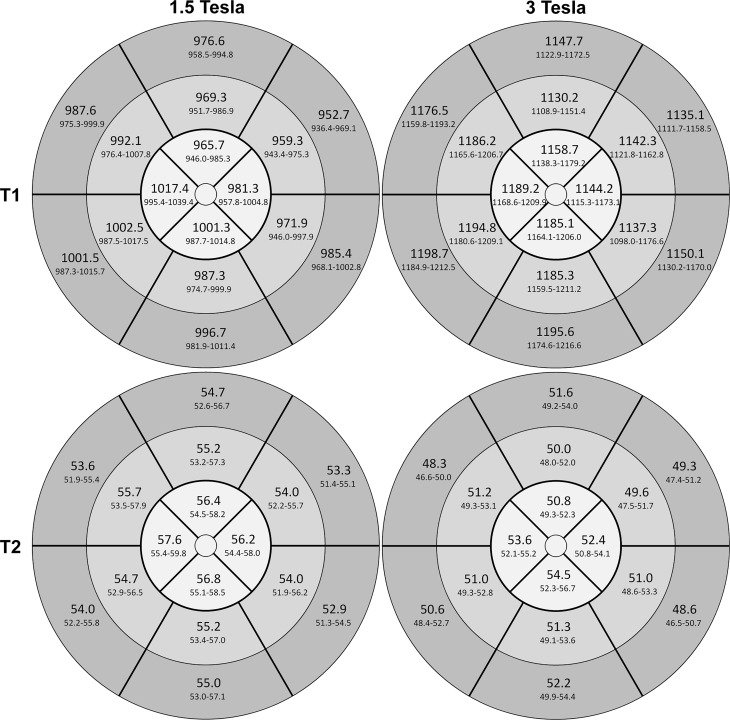
16-segment heart model (AHA), bull’s-eye plot of the left ventricle. Mean T1 and T2 relaxation time in milliseconds per segment plus 95% CI for males at 1.5T (*left*) and at 3T (*right*)

In addition, since sex-specific significant differences were revealed, gender-specific bull’s-eye plots were prepared. These are presented in Figs. 7 and 8 separately for females and males. Independently of gender, the lateral free wall showed lower T1 values than the septal region.

**Fig. 9:**
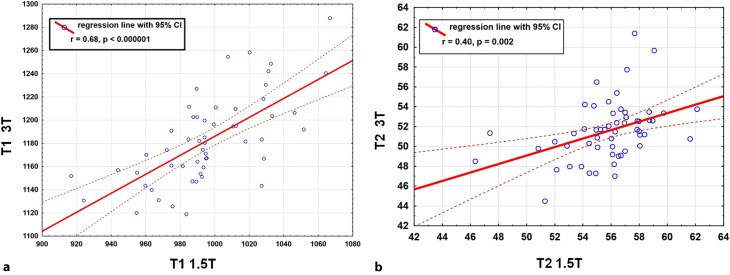
Correlation of global T1 relaxation times in milliseconds for 1.5T and 3T (**a**) illustrates a good correlation (r = 0.68, *p* < 0.000001), T2 values (**b**) show a moderate but still significant correlation (r = 0.4, *p* = 0.002), *dotted red lines* indicate 95% confidence intervals for regression line

Finally, the intra-individual correlation of T1 relaxation times independent of field strength in each volunteer was investigated and a significant correlation of intra-individual global T1 values measured at 1.5T and 3T (r = 0.68 and *p* < 0.001) could be confirmed (Fig. 9a).

### T2 mapping

At 1.5T a total of 897 segments were available, of which 57 were excluded due to artifacts (6.4%). At 3T 131 segments out of 907 segments were excluded due to artifacts (14.4%). The majority of excluded segments were in the basal slice, especially in the inferior and the lateral wall (Fig. 3). The same analysis was then performed as already described for T1 mapping. Mean global T2 relaxation times at 1.5T and 3T are presented in Table 2. According to published data [13, 20–22] T2 values were higher at 1.5T as compared to 3T (approximately 4.2 ms). Similar to T1 mapping, age and BMI did not influence T2 relaxation times (Table 3). Comparable to T1 mapping, females showed higher T2 values than males throughout all segments at 1.5T; however, at 3T the prolongation of global T2 relaxation in females was not significant (Table 3; Fig. 4b).

Mean relaxation times of the cohort per slice are shown in Table 4. The T2 relaxation times for 1.5T and 3T varied considerably depending on the slice, the values were highest in the apical slice and lowest in the basal slice. Significant differences of T2 values at 1.5T between basal and middle slices (*p* = 0.019), basal and apical slices (*p* < 0.001) and moreover between middle and apical slices (*p* < 0.001) were present. Moreover, significant differences between T2 values at 3T of basal and apical slices (*p* < 0.001) and middle and apical slices (*p* < 0.001) were evident. Gender-specific values of T2 relaxation times are presented in a 16-segment model of the heart (Figs. 7 and 8). Similar to T1 a significant per segment correlation of T2 values was revealed (r = 0.82, *p* < 0.001) (Fig. 6b) showing highest values in the apical region.

Heart rate seemed to have an effect on T2 relaxation times. A significant reduction (*p* = 0.02) of global T2 relaxation times by 2.0 ms was revealed at 3T in the subgroup of volunteers with a heart rate of ≥70 bpm. At 1.5T the reduction of T2 relaxation times was not significant (Table 3). Mean values plus normal range for T2 relaxation times per segment of subjects with heart rates below 70 bpm and with 70 bpm and above are illustrated in Fig. 5b for 1.5T and 3T.

As described for T1 mapping a statistically significant correlation of intra-individual global T2 values measured at 1.5T and 3T after scanning each proband on both scanners was also found (r = 0.40 and *p* = 0.002) (Fig. 9b).

## Discussion

This study was designed to establish reference values for T1 and T2 relaxation times in healthy volunteers for our specific setting at 1.5T and 3T. Furthermore, the correlation between the intra-individual global T1 and T2 values at different field strengths was studied and published data concerning possible influences of physiological factors on relaxation times were reproduced.

As already pointed out in previous publications, different mapping techniques and field strengths are associated with a variety of normal values [20]. These values were comparable with publications using similar mapping protocols showing an overlap in confidence intervals; however, differences were also observed even in comparison with studies using MOLLI techniques for T1 and GraSE techniques for T2 mapping which were applied in this cohort ([20]; Table 2). As indicated by Kellman and Hansen [23] reasons for this inconsistency might be due to small variations in the study protocols (e. g. different flip angle, differences in echo-spacing leading to partial volume errors, shimming, different MOLLI scheme). This has an important implication for clinical practice as apparently minor changes in mapping protocols might have an extensive impact on relaxation times. A recent study of Roy et al. [24] providing normal values using a 3T scanner from the same manufacturer but with a different MOLLI scheme with a 3(3)3(3)5 bSSFP sequence showed considerably lower T1 relaxation times of the whole heart than this study using a 5(3)3 bSSFP scheme (1122 ± 57 ms vs. 1184 ± 38 ms). This observation underlines that reference values have to be reassessed individually in every radiological department before implementing mapping techniques for routine CMRI examinations. The authors propose that a calibration of mapping protocols is necessary if myocardial T1 and T2 relaxation times are to be used as MRI biomarkers in clinical practice.

Apart from differences in study protocols patient-related physiological factors are also presumed to affect relaxation times. Of note, various studies found an association between aging and lower T1 and higher T2 relaxation times [17, 21, 25]. In this institute more than two thirds of the CMRI clientele are below the age of 60 years. To avoid age-related alterations, this study focused on a younger population (only 4 subjects above the age of 60 years with a mean age of 41 years, ranging from 20 to 69 years). Consistently, in this cohort no significant differences of T1 and T2 values between age groups (<45 years of age vs. ≥45 years of age) were found. Nevertheless, besides age gender and heart rate have also been discussed controversially as possible influencing factors for relaxation times [13, 21, 25, 26].

In this cohort, gender was a confounding factor for T1 and T2 relaxation times at 1.5T and 3T. According to previous studies, females showed a significant prolongation of T1 relaxation times at 1.5T and 3T and of T2 relaxation times at 1.5T [17, 26–28]; however, in accordance with von Knobelsdorff-Brenkenhoff et al. and Roy et al. [21, 24] no significant prolongation of T2 relaxation times in females at 3T was observed which might be associated with numerous artifacts at 3T in this study (Fig. 3). Furthermore, a heart rate ≥70 bpm led to a significant global prolongation of T1 relaxation times at both field strengths. Even though the MOLLI 5(3)3 schema was used which is supposed to be less sensitive to higher heart rates plus a frequency adapted algorithm for higher heart rates, an effect seems to remain [29]. A possible explanation might be the influence of partial volume effects due to relatively thick slices (8 mm) and cardiac motion artifacts especially in thin myocardial walls [23].

The prolongation of T1 relaxation times in higher heart rates is in accordance with Piechnik et al. [26] while others described no significant influences [21]. In accordance with previous studies a heart rate of ≥70 bpm was associated with a significant reduction of T2 relaxation times at 3T [21]. The small but significant influence of heart rate on T1 relaxation times and on T2 relaxation time at 3T leads to the assumption that an algorithm for compensation of higher heart rates is recommended to improve mapping quality for detection of subtle myocardial changes.

While a correlation of relaxation times at different field strengths in the same individual is presumed, to the best of our knowledge no study thus far has investigated this issue in a larger cohort. Therefore, we focused on this interesting subject and indeed were able to verify this association. This means that if a subject had low mean T1 values at 1.5T, these values were also low at 3T. As there are limited data on the reproducibility of myocardial T1 and T2 relaxation times at different field strengths in the same individual this is an important finding. The correlation was higher for T1 than for T2 values (Fig. 9). This might be associated with artifacts affecting T2 measurements especially at 3T, primarily off-resonance artifacts in regions adjacent to the lungs in addition to other artifacts, such as partial volume and motion artifacts. Of note, this is a critical point that becomes more relevant when evaluating the same individual at different field strengths. According to other publications, myocardial T1 relaxation times vary significantly with field strength. An increase of T1 values and a decrease of T2 values with higher field strength is reflected in previous studies [20, 28, 30–34]. The mean T2 value at 1.5T using a GraSE technique is consistent with other publications [4, 13, 22, 24, 35]; however, while at 3T the T2 relaxation time was comparable with Baessler et al. using a GraSE sequence as applied in this study, other authors described considerably lower T2 values using accelerated multi-echo spin echo (MESE) or T2-prepared bSSFP sequences [21, 36–38]. Again, this finding highlights the necessity of individual evaluation of normal values in every radiological institution. Furthermore, one can speculate that if institutional reference values are not available, only critical comparison with published relaxation times using similar protocols may be considered.

As already noted by other authors, also in zjis cohort the intrasubject and intersubject variability of T1 and especially of T2 values was considerably high per slice and per segment ([4, 21, 38], Table 4; Figs. 5, 6 and 7). This suggests a broad spectrum of normal values probably due to different effects of artifacts (e.g. B0, B1 inhomogeneities, off-resonance artifacts, motion artifacts, susceptibility artifacts or partial volume) in different areas of the myocardium. Apart from artifacts it has to be considered that e. g. regional differences in extracellular volume (ECV), amount of collagen, collagen fiber orientation and regional perfusion influence normal values. This insight complicates the distinction between borderline and pathologic relaxation times.

Due to respiratory or cardiac motion artifacts and susceptibility artifacts, a proportion of segments had to be excluded from this analysis (Fig. 3). Of note, the T2 maps at 3T emerged as the most vulnerable sequence. In contrast to Baessler et al. fewer visual artifacts were obtained using a comparable GraSE sequence at 1.5T but exclusion rate at 3T was almost the same (6.4% vs. 20–24% at 1.5T and 14.4% vs. 13–15% at 3T) [38]. The main cause of artifacts are B0 and B1 inhomogeneities which are especially problematic at 3T leading to differences in the signal across images and between segmental measurements. Especially while using thick slice imaging partial volume effects cause artifacts particularly in apical slices and in hypermobile regions, such as the free lateral wall. Interestingly, as previously described [38], also in this study the elimination of segments affected by artifacts did not lead to exclusion of extreme value outliers.

In this study the topics of extracellular volume (ECV) and post-contrast T1-mapping were not elaborated, as it was pointed out in previous studies that native T1-mapping could differentiate between healthy and diseased myocardium with high sensitivity, specificity and diagnostic accuracy [8, 39]. Even though noninvasive quantification of myocardial ECV via post-contrast T1-mapping is well validated [40, 41], native T1-mapping represents an excellent, rapid, noncontrast alternative for the detection of myocardial tissue remodelling [42]. Furthermore, The MOLLI 5(3)3 scheme used in this institution is known to have an excellent precision especially for native T1-mapping but is not optimal for shorter T1 values associated with contrast [23].

There are several limitations to this study. This was a single center study of moderate sample size using MRI scanners from only one manufacturer. Therefore, a center-specific and manufacturer-specific bias cannot be excluded. No histological examination of the myocardium was performed. In the post-processing phase no program for motion correction was available which led to higher exclusion-rates of myocardial segments. Despite the relatively small size of the cohort results of studies using similar mapping techniques could be reproduced. This underlines the validity of these reference values and implications. As specified above, two thirds of the patients undergoing CMRI evaluation were below the age of 60 years. Therefore, this cohort consisted of younger volunteers, which might obscure a pre-described age dependency of relaxation times in the aged myocardium. These normal values can only be applied and generalized in younger patients in institutions using similar mapping techniques to ours.

## Conclusion

The results obtained during daily clinical practice in healthy volunteers indicated that T1 and T2 relaxation times showed gender and heart rate dependencies, even in T1 maps using a less heart rate sensitive MOLLI variant. Due to artifacts caution should be applied especially while performing T2 mapping at 3T and while measuring relaxation times in the inferolateral wall. As already pointed out in the consensus statement by the Society for Cardiovascular Magnetic Resonance (SCMR) endorsed by the European Association for Cardiovascular Imaging (EACVI) [14], we also highlight that reference values have to be reassessed in every radiological institution before implementing mapping protocols in daily clinical practice. Furthermore, to the best of our knowledge, the presumed correlation of intra-individual global T1 and T2 values measured at 1.5T and 3T could be demonstrated for the first time in a larger cohort.
